# Draft Genome Sequence of Lactobacillus oris BE7N, Isolated from a Healthy Centenarian in Hainan, China

**DOI:** 10.1128/mra.00062-23

**Published:** 2023-04-04

**Authors:** Congyong Li, Yiming Zhao, Yinghui Zhang, Zhuanyu Li, Gang Sun

**Affiliations:** a Sixth Health Care Department, Second Medical Center of PLA General Hospital, Beijing, China; b Department of Gastroenterology and Hepatology, Hainan Hospital of PLA General Hospital, Sanya, Hainan, China; c Beijing QuantiHealth Technology Co., Ltd., Beijing, China; d Department of Gastroenterology and Hepatology, First Medical Center of PLA General Hospital, Beijing, China; University of Maryland School of Medicine

## Abstract

We present the draft genome sequence of Lactobacillus oris strain BE7N, which was isolated from a healthy male centenarian in Hainan, China. The final 2,129,000-bp draft genome has a G+C content of 50.05%. The genome was sequenced using paired-end Illumina sequencing.

## ANNOUNCEMENT

*Lactobacillus* species have been isolated from humans (1). However, few have been isolated from an extremely long-lived human. Lactobacillus oris has great probiotic properties and the ability to survive under human gastrointestinal conditions (2, 3). Here, we present the draft gene sequence of *L. oris* strain EB7N from a healthy centenarian.

We isolated *L. oris* strain BE7N from a fecal sample from a 104-year-old male subject from Sanya, Hainan, China (4). Written informed consent was obtained, and this study was approved by the Medical Ethics Committee of Hainan Branch of PLA General Hospital (no. 301hn11-2017-03).

*L. oris* strain BE7N was isolated from a healthy centenarian’s fecal sample according to a method described previously (4). Colonies were picked, restreaked to purity, and identified using matrix-assisted laser desorption ionization–time of flight mass spectrometry (MALDI-TOF MS). Then, all of the strains were stored at −80°C at Beijing Quantitative Health Technology Co., Ltd. We obtained *L. oris* BE7N as a freeze-dried sample from the company. The strain was revived at room temperature and streaked for single colonies onto solid MRS medium (Beijing Landbridge Technology Co., Ltd.) for 24 h at 37°C under anaerobic conditions (10% H_2_ plus 10% CO_2_ plus 80%N_2_). A colony was transferred into MRS liquid medium under anaerobic conditions at 37°C for 24 h, and the bacterial culture was obtained. After that, the culture was centrifuged at 12,000 rpm for 10 min and the supernatant was discarded. The pellet was washed once with phosphate-buffered saline (PBS). Bacterial DNA was extracted by using the DNeasy PowerSoil Pro kit.

The genome sequencing of *L. oris* strain BE7N was performed in Beijing Quantitative Health Technology Co., Ltd. (Beijing, China). Total DNA quality was measured by a spectrophotometer and with 1% agarose gel electrophoresis. Then, the DNA was subjected to library construction using KAPA HyperPlus PCR-free kit and sequenced on the Illumina Novaseq 6000 platform following the manufacturer’s instructions for 150-bp paired-end reads, producing ~1,149 Mbp of raw data.

Cutadapt software (5) (v.1.14, -m30) was used to remove the sequencing adapter. We filtered out low-quality reads with a Phred quality score of <20 or short reads of <30 bp with the SolexaQA package (6). Afterward, the remaining reads were aligned to the host genome using SOAPaligner (v.2.21, -M 4 -l 30 -v 10) (7) to remove the host read contamination, and 910 Mbp of raw data was obtained.

MetaWRAP (v.1.2) (8) was used to extract a high-quality draft genome from clean reads by *de novo* assembly and binning. GTDB-Tk v.1.5.0 (9), based on the Genome Taxonomy Database (GTDB) (10), was performed to assign taxonomic classifications to the genome. Assembly quality, determined by CheckM v.1.1.3 (11), showed that the integrity was 99.45% and the contamination was 0.00%. The total size of draft EB7N assembly was 2,129,000 bp, distributed into a G+C content of 50.05% and 62 contigs with an *N*_50_ value of 57,641 bp. By using PGAP (v.6.3) (12), the genome contains 2,075 putative coding sequences and 52 putative RNA genes.

### Data availability.

The genome was deposited under GenBank accession no. JAPEJB000000000. All data are available under BioProject accession no. PRJNA896692 and BioSample accession no. SAMN31562624. The raw reads have been deposited in the NCBI SRA database under accession no. SRX18537384.
